# FSD-C10, a Fasudil derivative, promotes neuroregeneration through indirect and direct mechanisms

**DOI:** 10.1038/srep41227

**Published:** 2017-01-23

**Authors:** Yan-Hua Li, Chong Xie, Yuan Zhang, Xing Li, Hai-fei Zhang, Qing Wang, Zhi Chai, Bao-guo Xiao, Rodolfo Thome, Guang-Xian Zhang, Cun-gen Ma

**Affiliations:** 1 Institute of Brain Science, Datong key Laboratory of Molecular and Cell Immunology, Shanxi Datong University, Datong, 037009, China; 2 Department of Neurology, Thomas Jefferson University, Philadelphia, PA 19107, USA; 3 “2011” Collaborative Innovation Center/Research Center of Neurobiology, Shanxi University of Traditional Chinese Medicine, Taiyuan 030024, China; 4 Institute of Neurology, Huashan Hospital, Institutes of Brain Science and State Key Laboratory of Medical Neurobiology, Fudan University, Shanghai, 200025, China

## Abstract

FSD-C10, a Fasudil derivative, was shown to reduce severity of experimental autoimmune encephalomyelitis (EAE), an animal model of multiple sclerosis (MS), through the modulation of the immune response and induction of neuroprotective molecules in the central nervous system (CNS). However, whether FSD-C10 can promote neuroregeneration remains unknown. In this study, we further analyzed the effect of FSD-C10 on neuroprotection and remyelination. FSD-C10-treated mice showed a longer, thicker and more intense MAP2 and synaptophysin positive signal in the CNS, with significantly fewer CD4^+^ T cells, macrophages and microglia. Importantly, the CNS of FSD-C10-treated mice showed a shift of activated macrophages/microglia from the type 1 to type 2 status, elevated numbers of oligodendrocyte precursor cells (OPCs) and oligodendrocytes, and increased levels of neurotrophic factors NT-3, GDNF and BDNF. FSD-C10-treated microglia significantly inhibited Th1/Th17 cell differentiation and increased the number of IL-10^+^ CD4^+^ T cells, and the conditioned medium from FSD-C10-treated microglia promoted OPC survival and oligodendrocyte maturation. Addition of FSD-C10 directly promoted remyelination in a chemical-induced demyelination model on organotypic slice culture, in a BDNF-dependent manner. Together, these findings demonstrate that FSD-C10 promotes neural repair through mechanisms that involved both immunomodulation and induction of neurotrophic factors.

Multiple sclerosis (MS) is a chronic inflammatory debilitating disease in the Central Nervous System (CNS) that affects over 2 million people worldwide. Oligodendrocyte death is believed to be essential in the pathogenesis of MS as CNS myelin is produced by oligodendrocytes, and the loss of these cells results in demyelination, axonal damage and severe impairment of neurological function^1^,^2^,^3^,^4^,^5^. Concurrently with inflammation and demyelinating processes, repair mechanisms are initiated in primary demyelinated lesions. Extensive remyelination can be observed during the early stage of MS by recruitment, proliferation and differentiation of oligodendrocyte precursor cells (OPC)^5^. However, the remyelination is reduced after successive relapses and failure of effective remyelination in progressive MS lesions is associated with a lack of oligodendrocyte maturation^6^,^7^ and increased axonal degeneration^8^. Therefore, stimulation of remyelination through an increase in oligodendrocyte maturation in the CNS lesions is critical to the functional recovery in MS^6^,^9^.

Fasudil, an inhibitor of Rho kinases (ROCK), has been shown to have beneficial effects on CNS-related disorders^10^,^11^. In EAE, Fasudil reduced the severity of disease through the stimulation of an anti-inflammatory response and a shift of M1 towards M2 macrophage/microglia^12^,^13^. M1 microglia secrete toxic molecules that destruct axon-supporting myelin and oligodendrocytes, whereas M2 cells release anti-inflammatory cytokines and growth factors that contribute to efficient remyelination and protect neurons from damage^5^,^14^,^15^. Manipulating the switch from M1- to M2-dominant polarization of microglia is a desirable strategy for efficient remyelination therapies. In addition, failure of spontaneous remyelination is also associated with a lack of sufficient amount of neurotrophic factors (BDNF, NT-3 and GDNF) in the CNS during inflammation^16^,^17^,^18^. In this context, our previous study showed that nasal administration of FSD-C10, a derivative of Fasudil with less toxic effect, effectively suppressed the clinical severity of experimental autoimmune encephalomyelitis (EAE), an animal model of MS. This effect was associated with a upregulated Tregs^19^. Still, whether FSD-C10 presents a neuroregenerative and neuroprotective effect has yet to be elucidated.

In the present study, we found that FSD-C10 significantly promoted neurological recovery, oligodendrogenesis, and remyelination. The mechanisms underlying these effects relayed on immunomodulation and direct neuroregeneration. Our data show that FSD-C10 has a beneficial effect on EAE acting through the modulation of the immune response and neuroregeneration.

## Results

### Intranasal FSD-C10 has a neuroprotective potential in EAE

**Figure 1:**
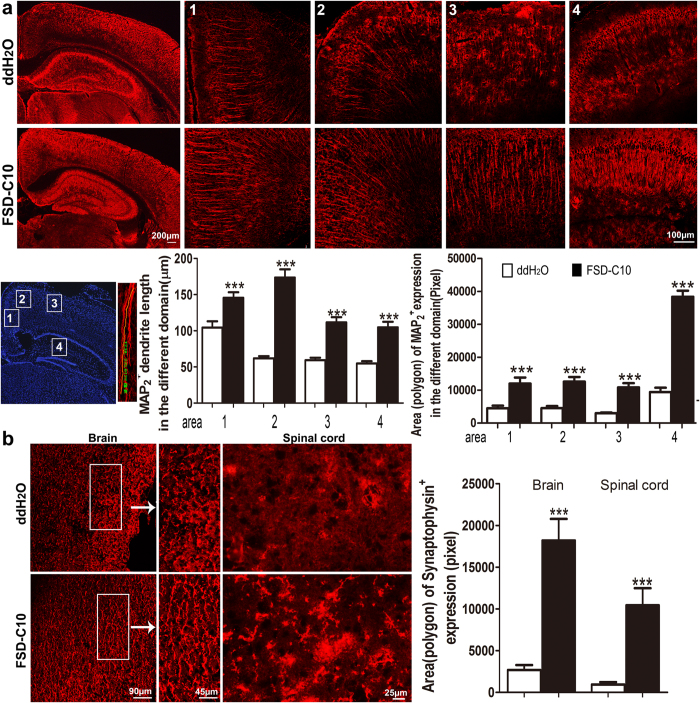
Intranasal FSD-C10 protects neuritis and synapses from damage in EAE. EAE was induced in C57BL/6 mice by immunization with MOG_35–55_/CFA. Mice received FSD-C10 or ddH_2_O by intranasal pathway beginning on day 3 p.i. until day 27 p.i. Brain and lumbar sections of spinal cord were harvested at day 28 p.i. and immunostained with MAP2 and synaptophysin. (**a**) MAP2 expression in the cortex (areas 1, 2 and 3) and hippocampal region (area 4) of brain. (**b**) Representative microphotographs and quantitative analysis for synaptophysin expression in the cortex of brain and the grey matter of spinal cord. Quantitative analyses represent mean ± SEM (n = 7 each group). ***P < 0.001. One representative of three experiments is shown.

Similar to our previous study^19^, nasal administration of FSD-C10 effectively suppressed clinical severity of EAE, with reduced CNS inflammation and demyelination (Figure S1). Extensive CD4^+^ T cells and CD68^+^ macrophages were found in brains from untreated EAE mice whereas the frequency of these cells were significantly reduced in mice treated with nasal FSD-C10 (Figure S2). In order to study the neural protection effect of FSD-C10, we treated MOG_35–55_-immunized mice with FSD-C10 (2.5 mg/kg/d). Treatment regimen started from day 3 p.i. until day 27 p.i. At the end of treatment, mice were euthanized and the CNS tissue was collected and analyzed for the expression of microtubule-associated protein 2 (MAP2), which is specifically expressed in dendrites and plays a key role in dendritic outgrowth, branching and synaptogenesis^20^,^21^,^22^. Our data show that FSD-C10 increased MAP2 expression and improved MAP2 positive dendritic morphology in prefrontal cortex and hippocampus compared to tissue from untreated EAE mice (Fig. 1a). Similar results were also observed in spinal cord tissues (data not shown). We further examined the synaptic structure in brain and the spinal cord by analyzing the expression of synaptophysin, a protein found in presynaptic vesicles. This parameter allows a general analysis of spinal cord circuit integrity^23^,^24^. The synaptophysin positive staining in the cortex of brain and the grey matter of spinal cord of FSD-C10-treated mice was significantly higher than in the control mice (Fig. 1b). These results indicate that FSD-C10 performed a neuropreservation in the CNS of EAE mice.

### Characterization of proliferative cells in the CNS of FSD-C10-treated mice

**Figure 2:**
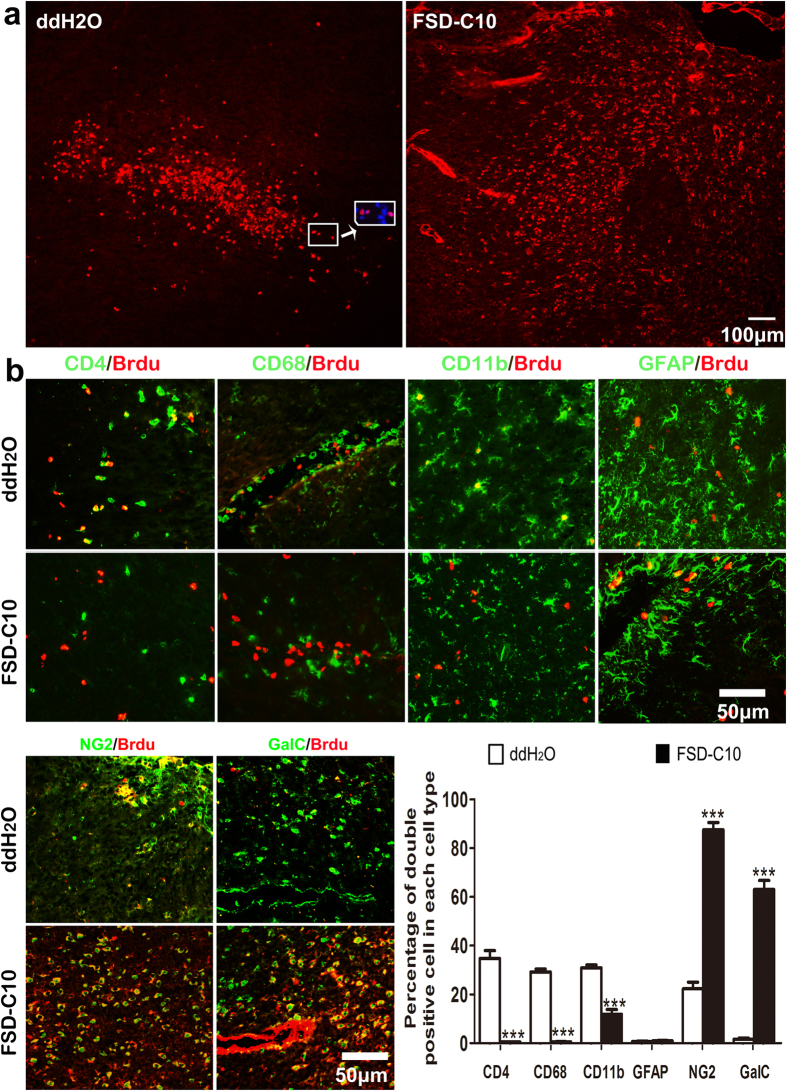
FSD-C10 stimulates the proliferation of OPCs and oligodendrocytes while suppresses proliferation of immune cells. Mice from each group received three i.p. injections of BrdU (sigma) on days 25–27 p.i. and brains were harvested from mice described in Fig. 1. (**a**) Proliferating cells were identified by a mouse anti-BrdU antibody. (**b**) Immunostaining of BrdU (red) and cell-specific markers (green) including CD4, CD68, CD11b, NG2 and GalC were performed. Representative microphotographs of double positive cells for BrdU and different cell types were shown, and percentages of these double positive cells in each cell type were analyzed. Data represent mean ± SEM (n = 7 each group). ***P < 0.001. One representative of three experiments is shown.

To study the mechanism underlying the neuroprotective effect of FSD-C10 in EAE, we first explored and characterized cell proliferation in the CNS after treatment. Mice received 3 i.p. injections of BrdU at days 25–27 post immunization (p.i.) and were euthanized at 28 days p.i. Analysis of coronal brain sections showed that, while the BrdU^+^ proliferative cells were mainly concentrated in perivascular space in tissue from untreated mice, these cells were extensively detected in the whole brain parenchyma of FSD-C10-treated EAE mice (Fig. 2a).

We then analyzed the proliferative status of different cell types in the CNS after FSD-C10 treatment. In ddH_2_O-treated EAE mice, 30–40% of immune cells, e.g., CD4^+^ T cells and CD68^+^/CD11b^+^ microglia/macrophages, were BrdU^+^, while the percentages of BrdU^+^ cells were significantly decreased by FSD-C10 treatment. In contrast, only low numbers of BrdU^+^ were colocalized with GFAP^+^ (astrocytes), NG2^+^ (OPCs) and GalC^+^ (mature oligodendrocytes) in ddH_2_O-treated EAE mice, and FSD-C10 treatment largely increased the proportions of BrdU^+^/NG2^+^ and GalC^+^ cells (Fig. 2b). Thus, FSD-C10 treatment effectively stimulated the proliferation of OPC/oligodendrocyte lineage cells, but suppressed proliferation of immune cells.

### Elevated numbers of OPCs and oligodendroglia in FSD-C10-treated mice

**Figure 3:**
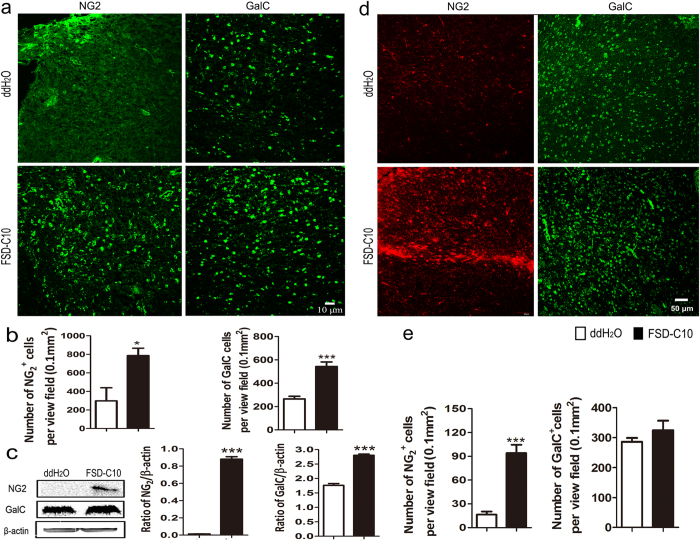
FSD-C10 increased the numbers of OPCs and oligodendrocytes. EAE (**a,b,c**) or naïve mice (**d,e**) received FSD-C10 or ddH_2_O by intranasal pathway. Brains were harvested from mice described in Fig. 1. Immunohistofluorescence of OPCs (NG2^+^) and oligodendrocytes (GalC^+^) within brains were examined. Representative microphotographs (**a**,**d**) and quantitative analyses for NG2^+^ and GalC^+^ cells from ddH2O-treated and FSD-C10-treated EAE mice (**b,e**), as well as representative Western blot photos and their quantitative analysis (**c**) were shown. Data represent mean ± SEM (n = 7 each group for brain section staining, and n = 4 each group for Western blot). *P < 0.05, ***P < 0.001. One representative of three experiments is shown.

To evaluate the effects of FSD-C10 on OPC recruitment and oligodendrocyte maturation, immunohistofluorescence was carried out on brain section with antibodies to NG2 and GalC, as markers of OPCs and oligodendrocytes, respectively. A low number of NG2^+^ cells was observed in brain of EAE control mice, and a significantly increased number of these cells was observed in brain of FSD-C10-treated EAE mice (Fig. 3a and b). Similarly, about a 2-fold increase of GalC^+^ oligodendrocytes in the brain of FSD-C10-treated EAE mice was seen compared to those in the untreated mice (Fig. 3a and b). These results were further confirmed by analysis of total protein (Fig. 3c). In contrast, administration of FSD-C10 in naïve mice increased the number of OPCs, but not oligodendrocytes (Fig. d,e), indicating that FSD-C10 affects OPC lineage cells in the physiological condition, without disturbing the regulation of mature myelinating cells *in vivo*.

### Intranasal FSD-C10 stimulated the production of neurotrophic factors

**Figure 4:**
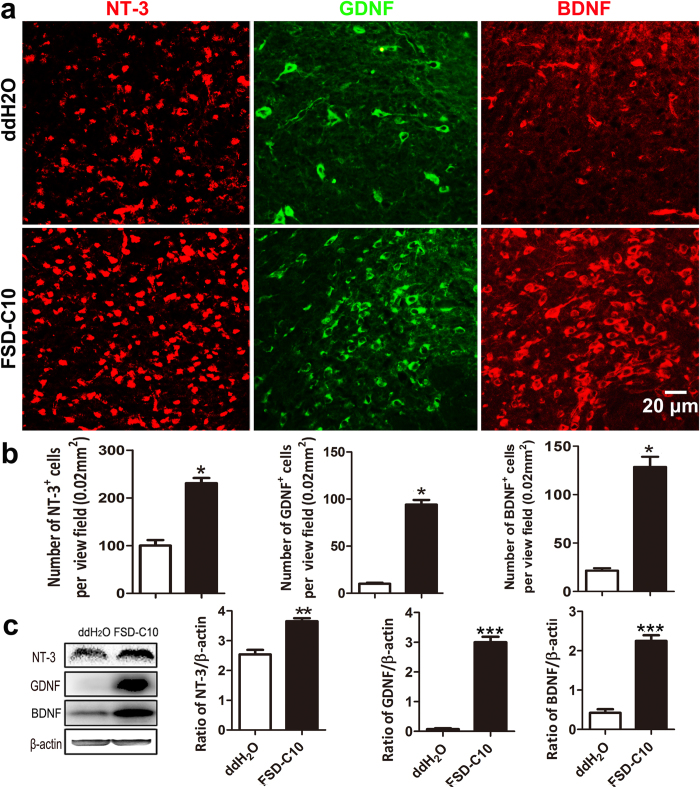
FSD-C10 stimulated the production of GDNF, NT-3 and BDNF in EAE. Brains were harvested from mice described in Fig. 1, and sections were immunostained with NT-3, GDNF and BDNF antibodies. Representative microphotographs (**a**) and their quantitative analyses (**b**) were shown. (**c**) Expression of these neurotrophic factors in brain tissues was also determined by Western blot and quantitatively analyzed. Data represent mean ± SEM (n = 7 each group for brain section staining, and n = 4 each group for Western blot). *P < 0.05, **P < 0.01, ***P < 0.001. One representative of three experiments is shown.

It was shown that induction of neurotrophins, including NT-3, GDNF, and BDNF, plays an important role in neurogenesis, remyelination, and brain repair^16^,^25^,^26^. We therefore determined if FSD-C10 treatment induced these neurotrophic factors. Our data show that while low numbers of cells producing these neurotrophic factors appeared in the brain of ddH_2_O-treated EAE mice, significantly increased numbers were observed in the brain of FSD-C10-treated EAE mice (Fig. 4a and b). Similarly, total protein analysis confirmed that a strong up-regulation of these neurotrophic factors was found in brain of FSD-C10-treated mice compared with EAE mice (Fig. 4c). These results indicate a stimulatory effect of FSD-C10 in the production of neurotrophic factors *in vivo*.

### Intranasal FSD-C10 shifted activated microglia from M1 to M2 status

**Figure 5:**
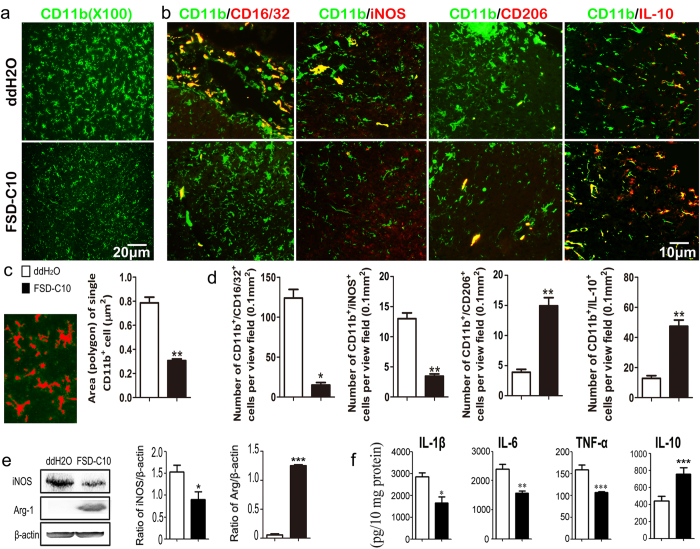
Intranasal FSD-C10 shifted inflammatory microglia into anti-inflammatory microglia. Brains were harvested from mice described in Fig. 1. (**a**) Brains were immunostained with CD11b antibody for analyzing the functional states and morphological diversity of microglia. FSD-C10 treatment reduced the numbers of fully ramified and triangle microglia (activated microglia). The single microglia area (polygon) was quantitatively analyzed by Image-pro plus 6.0 software in (**b**). (**c**) Expression of M1 markers (CD16/32 and iNOS) and M2 markers (CD206 and IL-10) in CD11b^+^ cells was determined by immunohistochemistry and (**d**) double positive cells were quantitatively analyzed. (**e**) Expression of iNOS and Arg-1 was also measured by Western blot. (**f**) Brains were homogenized on ice, and equal amounts of protein (10 mg) were measured by ELISA for cytokine production. Quantitative analyses represent mean ± SEM (n = 7 each group for brain section staining, and n = 4 each group for Western blot and ELISA). *P < 0.05, **P < 0.01, ***P < 0.001. One representative of three experiments is shown.

Given the important roles of different microglia/macrophages in CNS myelination, we then determined the functional states of microglia after FSD-C10 treatment. In normal brain, few ramified microglia (resting microglia) can be detected, while fully ramified microglia (activated microglia) are numerous in middle-injured brain; elongated microglia (triangles, phagocytic microglia) became rod cells, and probably fuse to form cell clusters in severe-injured brain^27^. In our experiment, numerous hyper-ramified and elongated microglia with large cell body were detected in brain of ddH_2_O treated EAE, indicating activated microglia. In contrast, microglia in FSD-C10-treated mice showed in a thin branched form (Fig. 5a and b).

Functionally, microglia can have different states, including the inflammatory and neurotoxic M1 and anti-inflammatory and neuroprotective M2 phenotypes^28^. Our results showed that, compared with control mice, FSD-C10-treated mice exhibited substantially reduced numbers of CD11b^+^ microglia/macrophages expressing the M1 markers iNOS and CD16/32, while numbers of these cells expressing the M2 markers IL-10 and CD206 were significantly increased (Fig. 5c and d). FSD-C10 inhibited the protein expression of iNOS while enhanced the levels of Arg-1 (Fig. 5e). Similarly, levels of M1 cytokines IL-1β, IL-6 and TNF-α were significantly suppressed while levels of IL-10 were upregulated after FSD-C10 treatment (Fig. 5f). These results indicate that intranasal administration of FSD-C10 blocked the microglia activation and switched these cells from a M1 to M2 phenotype. While FSD-C10 treatment in naïve mice increased Arg-1 expression in microglia, it did not affect the expression of iNOS and CD16/32, IL-10 and CD206 (Figure S3).

### FSD-C10 treatment inhibited Th1 and Th17 cells in EAE mice

**Figure 6:**
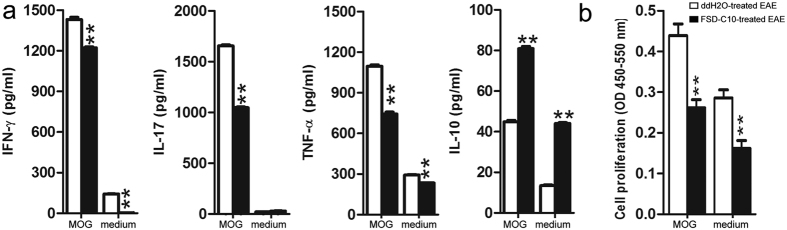
Immunomodulatory effects of intranasal FSD-C10 in the periphery. Splenocytes from each group of EAE mice (n = 3) were harvested on day 12 p.i., (**a**) *In vivo* inhibition of pro-inflammatory cytokines. Splenocytes were cultured at 5 × 10^6^ cells/ml and stimulated with MOG_35–55_ for 3 days. Cytokine production in supernatants was analyzed by ELISA. (**b**) Splenocytes were cultured at 2 × 10^6^ cells/ml in 100 μl/well and stimulated with or without MOG_35–55_ for 3 days and labeled with BrdU for cell proliferation assay by an ELISA reader following the manufacturer’s instructions (Abcam) (n = 8 each group). **P < 0.01, comparisons between ddH_2_O-treated animals and FSD-C10-treated groups. One representative of three experiments is shown.

We then explored whether intranasal administration of FSD-C10 could suppress the development of Th1 and Th17 cells. MOG_35–55_-immunized mice received ddH2O or FSD-C10 starting from day 3 p.i., their splenocytes obtained on 15 p.i., and treated with or without MOG_35–55_ (25 μg/ml) for 72 h. As shown in Fig. 6a, splenocytes of FSD-C10-treated EAE mice showed a lower production of IL-17, TNF-α, IFN-γ and a higher production of IL-10 than those of the PBS-treated group. In addition, FSD-C10 treatment also reduced MOG-induced T cell proliferation in the presence or absence of MOG (Fig. 6b).

### FSD-C10 shifted M1 to M2 phenotype *in vitro*

**Figure 7:**
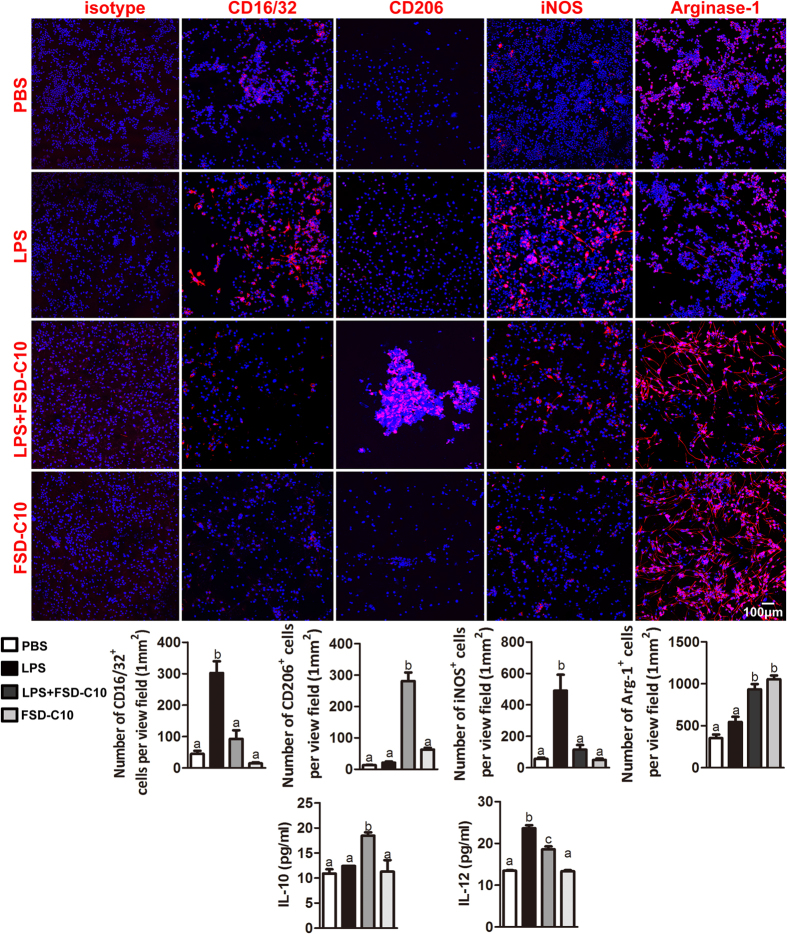
FSD-C10 shifted BV-2 microglia from M1 to M2 by immunostaining *in vitro*. BV-2 microglia were stimulated with PBS or LPS (0.5 μg/ml) in the absence or presence of FSD-C10 (15 μg/ml) for 24 h. Cells were stained with M1 microglia markers CD16/32, iNOS as well as M2 markers CD206, Arg-1. IL-12 and IL-10 were checked with ELISA. Representative photographs and quantitative analyses (mean ± SEM; n = 5 each group) from immunostaining and ELISA are shown. Results are expressed as number of immunostaining positive cells per view field and pg/ml. Groups designated by the same letter are not significantly different, while those with different letters (a,b,c) are significantly different (p < 0.05). One representative of three experiments is shown.

To test whether FSD-C10 treatment can directly induce a shift of M1 microglia towards a M2 phenotype, BV-2 microglia were firstly polarized to M1 by exposure to lipopolysaccharide (LPS), and then were treated with FSD-C10. The polarization was examined by immunofluorescence. Results showed that LPS stimulated high expression levels of M1 markers iNOS, CD16/32 and IL-12, but low levels of M2 markers CD206, IL-10 and Arg-1. FSD-C10 treatment significantly inhibited the expression of all M1 markers, but enhanced those M2 markers (Fig. 7). These results indicate a direct effect of FSD-C10 in the shift to the M2 phenotype in microglia.

### FSD-C10-treated microglia have a reduced ability to prime autoreactive T cells *in vitro*

**Figure 8:**
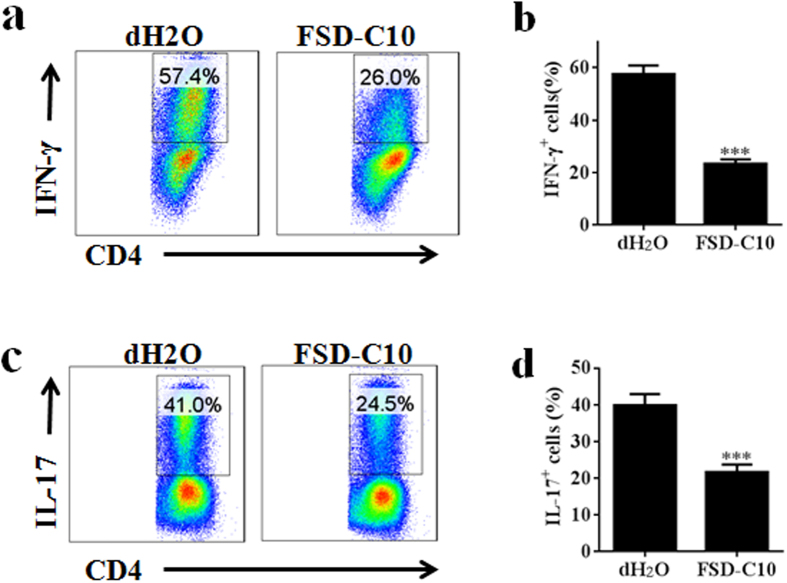
FSD-C10 modulated Th1 and Th17 cell differentiation. For differentiation of Th1 and Th17 cells, purified CD4^+^ T cells of normal female C57BL/6 mice (8 wks) were stimulated with anti-CD3 and anti-CD28 for 3 days under (**a,b**) Th1 polarization conditions (IL-12 and anti-IL-4) and (**c,d**) Th17 polarization conditions (TGF-β1, IL-6, IL-1β, anti-IL-4 and anti-IFN-γ) in the presence or absence of FSD-C10. Percentages of IFN-γ^+^ CD4^+^ Th1 cells and IL-17^+^ CD4^+^ Th17 cells were determined by flow cytometry. Data represent mean ± SEM (n = 5 each group). ***P < 0.001. One representative of three experiments is shown.

**Figure 9:**
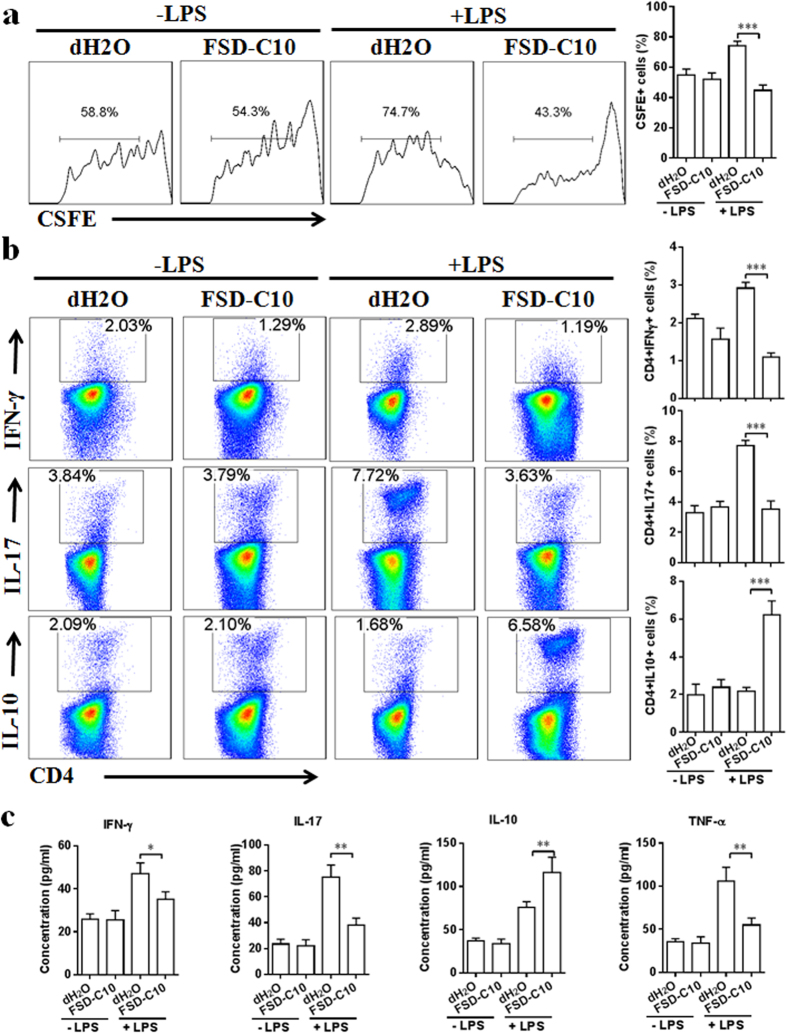
FSD-C10-treated microglia induce the conversion of MOG-reactive T cells *in vitro*. CD4^+^ T cells from EAE mice were labeled with CFSE and co-cultured with BV-2 microglia that were stimulated with or without LPS and treated with PBS- or FSD-C10 at an estimated ratio of 10:1, in the presence of MOG35–55 (25 μg/ml) for 72 h. (**a**) Percentage of proliferating cells was determined through the measurement of CFSE dilution using flow cytometry. Data represent mean ± SEM (n = 5 each group). (**b**) IFN-γ, IL-10, and IL-17 secretion was analyzed using flow cytometry. Data represent mean ± SEM (n = 5 each group). (**c**) The levels of IFN-γ, IL-10, IL-17, and TNF-α in culture supernatants were detected using ELISA kits. Data represent mean ± SEM (n = 8 each group). *P < 0.05, **P < 0.01, ***P < 0.001. One representative of three experiments is shown.

We then determined the direct effect of FSD-C10 on Th1/Th17 cells in purified CD4^+^ T cells, and showed that adding FSD-C10 *in vitro* significantly inhibited Th1/Th17 cell differentiation (Fig. 8). However, whether FSD-C10 can inhibit the capacity of microglia in priming effector T cell response remains unclear. To answer this question, we first determined the effect of FSD-C10-treated microglia on MOG-reactive T cell proliferation. While LPS-stimulated microglia promoted T cell proliferative responses, this capacity was inhibited when microglia were treated with FSD-C10 (Fig. 9a, P < 0.01). In contrast, microglia without LPS stimulation did not affect T cell proliferation (Fig. 9a).

Next, we examined whether polarization of the BV-2 microglia mediated by FSD-C10 could affect the population and function of the encephalomyelitic T cells. FSD-C10-treated BV-2 microglia were co-cultured with MOG-reactive T cells. As shown in Fig. 9b, FSD-C10-treated microglia following LPS maturation induced CD4^+^ IL-10^+^ T cells, whereas the percentages of MOG-reactive Th1 (CD4^+^ IFN-γ^+^) and Th17 (CD4^+^ IL-17^+^) cells were reduced. Consistent with the FACS data, ELISA assays confirmed that FSD-C10-treated BV-2 microglia inhibited IFN-γ, IL-17 and TNF-α production by T cells and induced them to produce IL-10, compared with the PBS-treated BV-2 microglia (Fig. 9c, P < 0.05–0.01). These results indicate that FSD-C10-treated microglia can convert the function of MOG-reactive T cells from pro-inflammatory to an anti-inflammatory one.

### LPS/FSD-C10-stimulated microglia cells produce factors that promote oligodendrocyte survival and maturation

**Figure 10:**
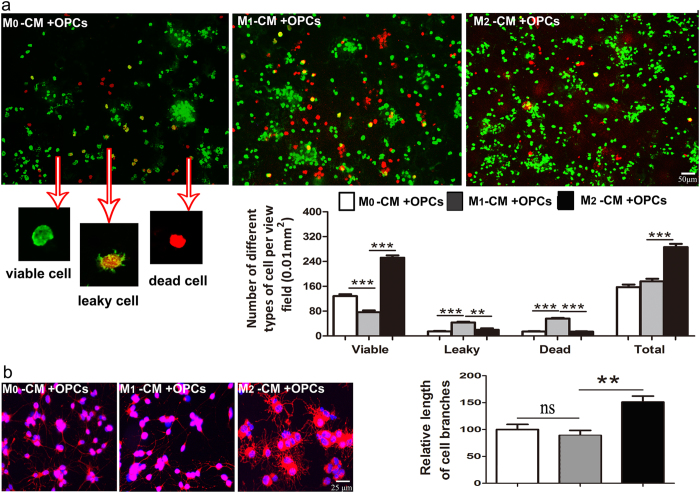
The CM from LPS/FSD-C10 stimulated microglia promoted OPC survival and oligodendrocyte maturation. OPCs were generated from brain of neonatal mice and cultured with conditioned media (CM) from microglia treated with PBS (M0), LPS (M1), or LPS^+^ FSD-C10 (M2) for 24 hrs. (**a**) OPCs were stained using Live/Dead staining kit as given in the material and methods section, and representative images of viable cells, dead cells, and leaky cells were shown. These cells were then counted and quantified under fluorescent microscopy by Image-Pro Plus software in a blinded fashion. (**b**) We added M0-CM, M1-CM and M2-CM to the OPC differentiation media in a 1:1 ratio and the OPCs were cultured for 7 days. Cells were then stained by anti-MBP antibody. Representative images and quantification of length of cell branches were shown, and measurements were done with Image-Pro Plus software. Data in this figure represent mean ± SEM (n = 5 each group). **P < 0.01; **P < 0.001. One representative of three experiments is shown.

We further explored the function of FSD-C10-treated microglia on OPC/oligodendrocytes. OPCs were incubated with conditioned media (CM) obtained from cultures where microglia cells were treated with PBS (M0), LPS (M1), or LPS/FSD-C10 (M2) as described above. After 24 h of incubation with CM, OPCs were stained with the Live/dead cell imaging kit to assess cell viability. The percentages of leaky OPCs were significantly lower in cells cultured with the M2-CM than those with M1-CM. The M1-CM also markedly induced OPC death, however, the CM from PBS- and LPS/FSD-C10-stimulated microglia exhibited a significantly lower death rate than the LPS treatment group (Fig. 10a). These results indicate that, in contrast to M1-CM, conditioned media from FSD-C10-treated microglia cells has no toxic effect over OPC.

We also studied the effect of different CMs on OPC maturation. After culturing OPCs with CM for 7 days, these cells were stained with MBP, a marker of oligodendrocytes. Although all 3 groups of OPCs expressed MBP, M2-CM-treated group had elevated multipolar branches, with a significant increase in arborization (Fig. 10b). Taken together, the addition of M2-CM not only promoted the OPC survival but also increased the arborization/maturation of oligodendrocytes.

### FSD-C10 directly promotes remyelination *in vitro* in a BDNF-dependent manner

**Figure 11:**
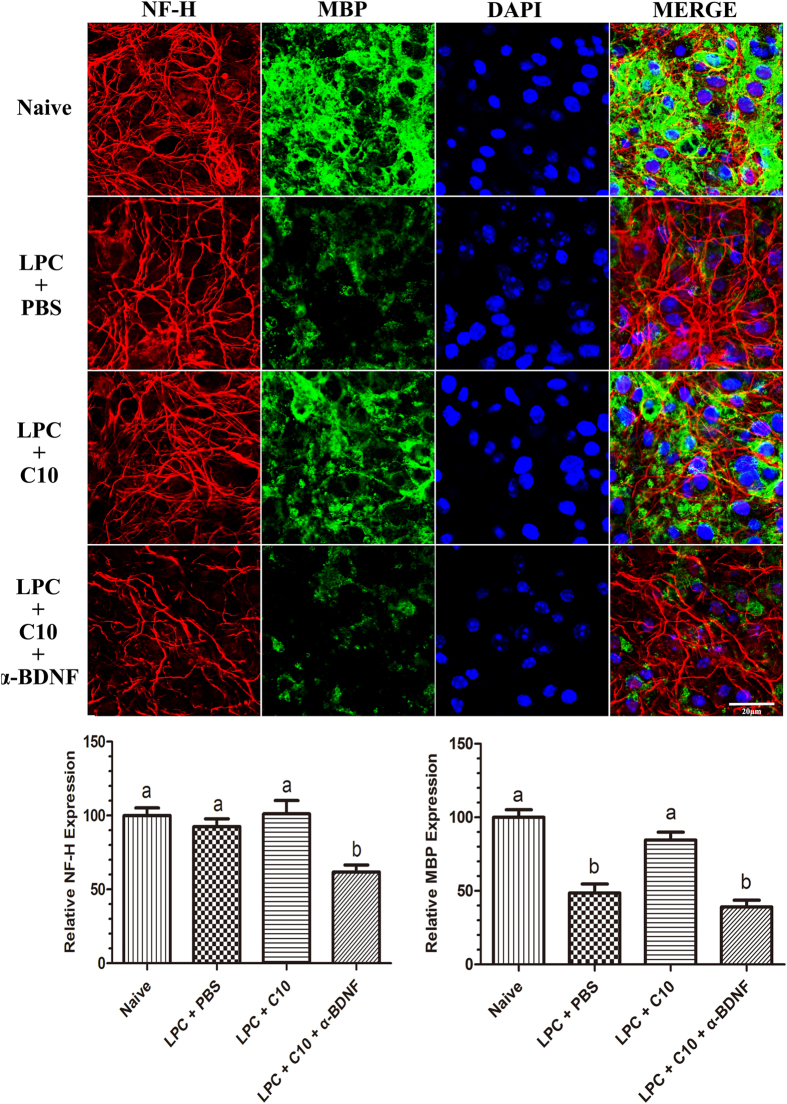
FSD-C10 directly promoted the formation of myelin sheath. Postnatal day 5 (P5) mouse pups were sacrificed and consecutive slices (350 μm) were cut from the cerebellums. Three slices in each 6-well insert plate were cultured in 1 ml medium. LPC was added into *cerebellum* slice cultures for 18 hrs to induce demyelination and then slices were cultured with 15 μg/ml FSD-C10 or PBS for 14 days, with or without antibodies to CNTF, NT-3 and BDNF. Slices were then stained with anti-MBP and anti-NF-H. Results were visualized by confocal microscopy and MBP and NF-H expression was analyzed by ImageJ software. Quantitative analyses represent mean ± SEM (n = 5 each group). Groups designated by the same letter are not significantly different, while those with different letters (a, b) are significantly different (p < 0.05). One representative of three experiments is shown.

To examine whether FSD-C10 can directly promote remyelination, a LPC-induced demyelination model in organotypic slice culture was conducted. In this experimental approach, demyelination is achieved after incubation of cultures with LPC for 18 h. Treatment with LPC leads to a decreased expression of MBP in organotypic cultures. When these demyelinated slices were treated with FSD-C10 for 14 days, MBP expression was significantly enhanced compared with the PBS-treated slices, suggesting a significant promotion in remyelination by FSD-C10 treatment (Fig. 11).

Given that FSD-C10 stimulated the production of the neurotrophic factors NT-3, GDNF and BDNF *in vivo* (Fig. 4), we aimed to evaluate whether these proteins played a role in FSD-C10-induced remyelination. For that matter, demyelinated organotypic cultures were treated with FSD-C10 in the presence of neutralizing antibodies against these factors. Our results showed that FSD-C10-induced remyelination was dramatically blocked by anti-BDNF antibodies (Fig. 11). Blockage of GDNF or NT-3 did not revert the effect of FSD-C10 (data not shown). Similarly, NF-H expression (neurons) was also largely reduced when anti-BDNF antibodies were added (Fig. 11). These results indicate that FSD-C10 can directly induce remyelination through a BDNF-dependent pathway.

## Discussion

In this paper we defined some of the mechanisms of FSD-C10-induced EAE amelioration. We found that FSD-C10 stimulates neurogrowth and regeneration, shifts macrophage/microglia activation profile and promotes remyelination. Inhibition of Rho associated kinase (ROCK) has been shown to promote OPC differentiation and myelin formation^29^,^30^,^31^,^32^. We have recently found that nasal administration of FSD-C10, a novel and efficient ROCK inhibitor, suppressed the activity and expression of ROCK, alleviated disease severity and CNS inflammation of EAE mice, and preserved myelin sheath^19^,^33^. Our data showed a higher proliferation rate of OPCs and mature oligodendrocytes in the CNS of FSD-C10-treated mice, which confirms the protective effect of FSD-C10 on CNS myelination *in vivo*. In a series of *in vitro* experiments, FSD-C10 treatment induced type 2 macrophages/microglia and the condition media of these cells protected OPCs from inflammation-induced death and promoted the maturation of these cells into oligodendrocytes. Further, addition of FSD-C10 into LPC-induced demyelination cultures effectively promoted remyelination in a BDNF-dependent manner.

MS/EAE is initiated through the activation of encephalitogenic myelin-responsive CD4^+^ T cells, which invade the CNS^1^,^2^. Together with lymphocyte infiltration, resident microglia are activated and present self-antigens to lymphocytes. A large proportion of circulating monocytes infiltrate the CNS as well. Monocytes/Macrophages and microglia are the ultimate effector cells in neuroinflammation, leading to the destruction of the myelin sheath and oligodendrocyte death^1^,^2^. Microglia cells play a pivotal role in the pathogeny of MS and EAE^34^. In EAE, activated microglia cells release inflammatory mediators, which lead to neurotoxic consequences. The existence of activated microglia is the most prominent feature of chronic neuroinflammation or neurodegenerative disease^35^. Our data show that FSD-C10 treatment significantly reduced the numbers of these immune cells in the CNS. Their proliferation in the disease foci was also significantly inhibited following treatment. These results indicate that FSD-C10 can alleviate EAE through a reduction in either the recruitment of peripheral immune cells as well as their proliferation in the CNS. We also found that FSD-C10 kept microglia in a thin branched form or possibly reverted the reactive microglia back to the resting state. In addition, intranasal FSD-C10 treatment significantly decreased microglia proliferation and their IL-1β and TNF-α expression. As a result, the cytotoxic outcome of these pathologic mediators on neural cells was significantly diminished.

In CNS, microglia undergo M1 or M2 polarization in response to different environmental stimuli^36^. For instance, while M1 microglia are typically found in of MS lesions and their frequency directly correlates with the extent of axonal damage, M2-polarized microglia contributes to regenerative response in the CNS^37^. Importantly, M2 microglia are lacking in chronic lesion of EAE^5^. Blockage of M2 profile in microglia has been shown to contribute to impaired remyelination^38^. Therefore, it is reasonable to speculate that stimulating the M2-polarized phenotype of microglia would confer protection from inflammation-induced neurodegeneration and demyelination. Our results show a clear M2 phenotype of macrophage/microglia after nasal FSD-C10 treatment. Interestingly, FSD-C10-treated microglia effectively induced IL-10-producing, but inhibited IFN-γ/IL-17 producing T cells, and conditioned media from M2 cultures increased the survival rate of OPCs and arborization/maturation of oligodendrocytes. Our observations suggest that FSD-C10 treatment induces M2 microglia, which have the capacity to enhance OPC survival and their maturation into oligodendrocytes.

It has been shown that loss of trophic support for oligodendrocytes and neurons leads to the failure of spontaneous remyelination and neural recovery in MS/EAE^39^. A treatment that has capacity to induce the production of neurotrophic factors in the disease foci would be greatly beneficial. Indeed, overexpression of NT-3 in neural stem cells reduces CNS inflammation and neurological deficits in ongoing EAE through the stimulation of proliferation and differentiation of neural stem cells into oligodendrocytes and neurons culminating in remyelination and neuronal repopulation^16^. Local release of NT-3 also reduces astrogliosis, a main cause of MS plaque formation^40^,^41^. GDNF, a member of the TGF-β superfamily^42^, promotes survival and differentiation of dopaminergic and motor neurons^43^,^44^, selectively enhances regrowth of damaged axons^45^, and induces myelin formation^18^,^46^. BDNF is well-known to promote neuronal survival and axonal growth^47^,^48^. BDNF also stimulates oligodendrocyte differentiation and maturation^49^,^50^, and protect the myelinated axons^51^,^52^. Although FSD-C10 treatment upregulated the production of NT-3, GDNF and BDNF, we found that blockage of BDNF abolished the beneficial effect of FSD-C10. Thus, BDNF plays an important role in FSD-C10-induced remyelination.

Our study showed that FSD-C10 is an efficient therapeutic drug for EAE. By inhibiting the migration and expansion of peripheral immune cells in the CNS, shifting macrophages/microglia from M1 to an M2 phenotype, and enhancing the production of neurotrophic factors, FSD-C10 demonstrated to exert several immune and non-immune beneficial effects in the course of neuroinflammation. Ultimately, FSD-C10 promoted remyelination and neuroprotection. Together, these findings demonstrate that FSD-C10 promotes neural repair in CNS autoimmunity through mechanisms involved both immunomodulation and induction of neurotrophic factors.

## Materials and Methods

### Mice

Female C57BL/6 mice (10–12 weeks and 18–20 g) were purchased from Vital River Laboratory Animal Technology Co. Ltd. (Beijing, China). All animal experiments were conducted in adherence with the International Council for Laboratory Animal Science guidelines. The study was approved by the Council for Laboratory and Ethics Committee of Shanxi Datong University, Datong, China. All mice were housed under pathogen-free conditions and kept in a reversed 12:12-h light/dark cycle in a temperature-controlled room (25 ± 2 °C) for one week prior to experimental manipulation.

### Induction of chronic EAE

EAE were induced as described previously^19^. Mice were subcutaneously immunized with 300 μg mouse myelin oligodendrocyte glycoprotein 35–55 peptide (MOG_35–55_, MEVGWYRSPFSRVVHLYRNGK) in Freund’s complete adjuvant (Sigma-Aldrich, St. Louis, MO, USA) supplemented with 3 mg/ml of Tuberculosis H37Ra (BD Difco, NJ, USA). In addition, 750 ng Pertussis toxin (Enzo Life Sciences, NY, USA) was injected intraperitoneally on days 0 and 2 p.i. MOG_35–55_ was produced in an automatic synthesizer (CL. Bio-Scientific., Xi’an, China). Purity of the peptide was > 95% as determined by HPLC.

### Administration of Fasudil derivative FSD-C10

MOG_35–55_ immunized mice were divided into 2 groups (n = 12 each group), and normal mice were also divided into 2 groups (n = 6 each group); all were treated with either FSD-C10 (FSD-C10-treated) or ddH_2_O (ddH_2_O-treated control). FSD-C10 is a Fasudil derivative that is easily soluble in ethanol, and suitably water-soluble. FSD-C10 (from Tianjin Chase Sun Pharmaceutical Co., Ltd, Tian’jin, China) was dissolved in sterile ddH_2_O. For intranasal administration, mice were slightly anesthetized with diethylether, and then received 10 μl/each nostril (2.5 mg/kg/day) of FSD-C10 at the opening of the nostrils on day 3 until day 27 p.i., allowing the animal to sniff the solution into the upper nasal cavity. Mice that received the same volume of ddH_2_O nasally served as untreated control.

### Cell proliferation

To study *in vivo* cell proliferation, mice received daily i.p. injections of BrdU (50 mg/kg) (Sigma) on days 25 until 27 p.i, and brain tissues were harvested at day 28 p.i. Proliferating cells were identified by using a mouse anti-BrdU antibody (1:500, Thermo Fisher Scientific, Waltham MA, USA) following experimental procedure recommended by the manufacturer. Briefly, brain sections were pretreated 10 min in paraformaldehyde (PFA) 4%, rinsed in PBS, and incubated 30 min in a solution of 2 N HCL. Slides were rinsed twice in PBS, and sections were incubated 10 min at 37 °C with the 0.1% trypsin. Slides were rinsed three times in PBS and then stained with anti- BrdU antibody.

### Tissue processing

Mice underwent intracardiac perfusion under deep anesthesia with saline and 4% PFA solution in PBS. Brains were removed, and incubated overnight in sucrose solution 10%, 20%, and 30% respectively at 4 °C. 10 μm coronal brain sections were collected on poly-lys-coated slides by leica cryotome (Leica, Rueil-Malmaison, France). Brain slices were cut (−0.82 to −2.54 mm posterior to bregma based on the paxinos atlas) for 170 sequential sections.

### Immunohistochemistry

Brain slides were air-dried, permeabilized, and blocked for 1 h and primary antibody applied overnight at 4 °C in a humid chamber. Fluorescently-conjugated secondary antibodies were applied for 2 h at room temperature in a humid chamber. Antibodies used to detect M1 microglia are as follows: mouse anti-iNOS (Enzo Life Sciences), rat anti-CD16/32 (BD Pharmingen, NJ, USA), anti-TNFα (Peprotech, NJ, USA), and IL-12 (Peprotech). Antibodies used to detect M2 markers are as follows: goat anti-Arginase-1 (anti-Arg-1, eBioscience, San Diego, CA, USA), IL-10 (Abcam, Camb, UK), CD206 (eBioscience). Antibodies against microglia/macrophage markers include rat anti-CD68 (Serotec, Kidlington, UK), CD4 (BD Pharmingen), CD11b (eBioscience). Oligodendrocyte, OPC neuron were detected by anti-NG2 (Promega, Madison, WI, USA), and GalC (Promega), anti-microtubule associated protein-2 (anti-MAP2, Millipore, Billericay, MA, UK) and anti-synaptophysin (Abcam). Other antibodies include rabbit anti-GFAP (Millipore), anti-NT-3 (Abcam), anti-GDNF (Novus Biologicals, CO, USA, 1:1000), anti-BDNF (Abcam). As a negative control, additional sections were treated similarly, but the primary antibodies were omitted. Results were visualized under fluorescent microscopy by Image-Pro Plus software or confocal laser scanning microscope (CLSM, Olympus, Tokyo, Japan) by FV-1200 software in a blinded fashion. Quantification was performed on three coronal sections per mouse, and six mice per group were analyzed.

### Western blot analysis

The brains were homogenized on ice with a Ultrasonic crusher (HD3000, Wiggens, Beijing, China) in ice-cold lysis buffer (1 × PBS, 1% Nonidet P-40, 0.5% sodium deoxycholate, and 0.1% SDS, RIPA) supplemented with protease inhibitors PMSF. Lysates were centrifuged at 10,000 × g for 20 min at 4 °C for two times, and the supernatants were collected. Protein concentrations were determined by a Bradford protein assay. Equal amounts of protein (30 μg) were separated by SDS-PAGE, and transferred onto a polyvinylidene fluoride filter (PVDF) membrane (Millipore). Membranes were blocked with 5% non-fat milk, and incubated at 4 °C overnight with anti-NG2, anti-GalC, anti-GDNF, anti-NT-3, anti-BDNF, anti-iNOS, anti-Arginase-1 and anti-β-actin (Cell signaling, Davers, MA, USA). Bands were visualized by HRP-conjugated secondary antibodies (Thermo Scientific, MA, USA) and chemiluminescence (ECL) kit under ECL system (Millipore).

### Cytokine measurement by ELISA

Levels of IL-1β, IL-6, TNF-α, IL-12, and IL-10 were measured by sandwich ELISA kits (Peprotech or R&D Systems, Minneapolis, MN) in accordance to manufacturer’s instructions. Determinations were performed in 3 independent experiments.

### Splenocyte proliferation and cytokine measurement *ex vivo*

Splenocytes were isolated from ddH2O-treated and FSD-C10-treated EAE mice on day 12 p.i., and cultured with or without 25 μg/ml MOG_35–55_ peptide for 3 days. Cell proliferation was determined by BrdU-incorporation test using BrdU Cell Proliferation ELISA Kit (Abcam). Supernatants from splenocytes were collected at 72 h to measure concentration of IFN-γ, IL-17, TNF-α, and IL-10 using ELISA kits (R&D System, Minneapolis, MN).

### Microglia polarization

The BV2 immortalized microglial cell line was obtained from ShenKe Biological Technolology CO., Ltd., Shanghai, PR China, and cultured in Dulbecco′s modified Eagle medium (DMEM; Gibco, Waltham MA, USA) supplemented with 10% fetal bovine serum (FBS; Gibco), 100 U/ml penicillin, and 100 μg/ml streptomycin (Gibco) at 37 °C in a humidified cell incubator with a 95%/5% (v/v) mixture of air and CO_2_. BV2 cells were allowed to attach to the culture dish for 2 h. Later, cells were either left untreated or treated overnight with lipopolysaccharide (LPS, Sigma-Aldrich, St. Louis, MO, USA, 500 ng/ml), FSD-C10 (15 μg/ml) or LPS plus FSD-C10. Conditioned media was collected and stored at −20 °C. Cells were acquired for immunostaining analyses.

### Th1/Th17 cell polarization of CD4^+^ T cells *in vitro*

Differentiation of Th1 and Th17 cells was induced *in vitro* following previously described protocols^53^. Briefly, single-cell suspensions derived from spleen of normal female C57BL/6 mice (6–8 week) were purified by negative selection with a mouse CD4^+^ T Cell Isolation Kit II (Miltenyi Biotec). Purified naïve CD4^+^ T cells were cultured with soluble anti-CD3e (5 μg/ml) and anti-CD28 (2 μg/ml) under their respective polarizing conditions. IL-12 (5 ng/ml) was added to induce differentiation into Th1 cells. TGF-β1 (2 ng/ml), IL-6 (20 ng/ml), IL-1β (10 ng/ml), anti-IL-4 (10 μg/ml), and anti-IFN-γ (10 μg/ml) were added in Th17 polarizing conditions. FSD-C10 at 15 μg/ml was also added to define its direct effect on Th1/Th17 cell differentiation, and cultures without FSD-C10 served as control. Cells were collected 3 days later for flow cytometric analysis.

### Co-culture of microglia and MOG-reactive T cells

Splenocytes were harvested from the spleen of EAE mice on day 8 p.i., and CD4^+^ T cells were purified using anti-mouse CD4 magnetic beads (Miltenyi Biotech, Auburn, CA, USA). These cells were then labeled with carboxyfluorescein diacetate succinimidyl ester (CFSE) for 20 min and washed for co-culture assay. The number of proliferating cells was determined through CFSE dilution with flow cytometry.

BV-2 microglia were cultured in the presence or absence of LPS (500 ng/ml) and/or FSD-C10 (15 μg/ml) for 24 h, washed, and then co-cultured with MOG-reactive CD4^+^ T cells prepared as described above at an estimated ratio of 1:10 (microglia: T cells) in the presence of MOG_35–55_ (25 μg/ml) for 72 h. Culture supernatants were collected to analyze cytokine production, and the cells obtained for flow cytometric analysis.

### Flow cytometry

Splenocytes were stimulated with 50 ng/ml PMA and 500 ng/ml ionomycin in the presence of GolgiPlug for 4 h. Cells were surface-stained with mAb against CD4. Cells were then washed, fixed, and permeabilized with Fix & Perm Medium (Invitrogen), and intracellular cytokines were stained with Abs against IL17, and IFN-γ, or IL10 (BD Biosciences). Flow cytometric analysis was performed on FACSAria (BD Biosciences, San Jose, CA) and data were analyzed with FlowJo software (Treestar, Ashland, OR).

### Immunocytochemistry staining

BV-2 cells were plated in 24 wells plates with coverslips. Cells were fixed with 4% PFA solution, washed in PBS for three times. Antibodies used to detect M1 microglia are as follows: anti-iNOS, anti-CD16/32. Antibodies used to detect M2 markers are as follows: anti-Arg-1, CD206. Results were visualized by confocal microscopy (FV1200; Olympus, Japan).

### OPC culture and conditioned medium treatment

OPCs were isolated from CNS tissue of mice using magnetic isolation beads according to manufacturer’s instruction (CD140a (PDGFRa), Miltenyi Biotec, Cologne, Germany) and cultivated as follows. 2 × 10^4^/ml OPCs were plated in 24-well plates with 1 ml DMEM/F12 containing 10 ng/ml PDGF AA, 20 ng/ml b-FGF, 2% N2, 2% B27, 1% p/o^29^,^54^. OPCs were treated with microglia media as conditioned media (CM) added in a 1:1 ratio to OPC culture media. After culturing for 24 h, viability of OPCs was assessed by using the Live/Dead^®^ Cell Imaging kit (Invitrogen, Cal, USA) in accordance with the protocol provided by the manufacturer. Twenty to 60 images were made from each sample and in each image the cells were counted. Cells with a green fluorescent cytoplasm and a dark nucleus were counted as “viable cells”, while cells with only a red nucleus were counted as “dead cells”. Cells that had a red nucleus with green cytoplasm, and were consequently in an intermediate state between viable and dead, were denoted as “leaky cells”. The sum number of viable cells, dead cells and leaky cells were defined as “total cells”^55^,^56^,^57^. Results were expressed as number of different type of cell per view field. Data are presented as mean ± SEM. To study the effect of conditioned media (CM) on OPC differentiation, we cultured OPCs in DMEM/DF12 medium containing 2% N2, 2% B27, 40 ng/ml triiodothyronine and 10 ng/ml ciliary neurotrophic factor (CNTF). We added CM to the differentiation medium in a 1:1 ratio and the OPCs were cultured for 7 days. Cells were then stained by anti-myelin basic protein (MBP, Abcam) antibody.

### FSD-C10 treatment of LPC-induced demyelination in cerebellum slice cultures

Postnatal day 5 (P5) mice pups were killed, and the cerebellums were removed and dissected in the dissection medium containing 50% HBSS, 50% Opti-MEM, following previously described protocol^58^,^59^. Consecutive slices (350 μm) were cut from the cerebellums using a tissue chopper. We cultured 3 slices in each 6-well insert plate (Millipore) with 1 ml medium composed of 25% heat-inactivated horse serum, 25% HBSS, 50% Opti-MEM. Demyelination was performed by adding 10 mg/ml lysophosphatidylcholine (LPC, Sigma-Aldrich) in the media on day 10. After culturing 12 h of adding LPC, slices were washed and transplanted to new inserts, and 15 μg/ml FSD-C10 or PBS was added to the culture media. To study the effect of neurotrophins on remyelination, neutralizing antibodies to GDNF, NT-3 and BDNF (Abcam, USA) were also added to the media. Slices were stained with anti-MBP and anti-NF-H on day 24 after culturing. Results were visualized by confocal microscopy (LSM 510; Zeiss, Jena, Germany).

### Statistical analysis

GraphPad Prism software was used for statistical analysis. Data are presented as mean ± SEM. Experiments were analyzed by Student’s *t*-tests, and *P*-values were considered statistically significant when *P* < 0.05.

## Additional Information

**How to cite this article:** Li, Y.-H. *et al*. FSD-C10, a Fasudil derivative, promotes neuroregeneration through indirect and direct mechanisms. *Sci. Rep.* **7**, 41227; doi: 10.1038/srep41227 (2017).

**Publisher's note:** Springer Nature remains neutral with regard to jurisdictional claims in published maps and institutional affiliations.

## Supplementary Material

Supplementary Information
