# Supplementary material for: FSD-C10, a Fasudil derivative, promotes neuroregeneration through indirect and direct mechanisms
Source: Sci Rep. 2017 Jan 23;7:41227. doi: 10.1038/srep41227 (PMC5255566; doi:10.1038/srep41227)
Supplement: Supplementary Information [file srep41227-s1.pdf]

FSD-C10, a Fasudil derivative, promotes neuroregeneration through indirect and direct mechanisms

Yan-Hua Li[a,1], Chong Xie[b,1], Yuan Zhang[b], Xing Li[b], Hai-fei Zhang[a], Qing Wang[c], Zhi Chai[c], Bao-guo Xiao[d], Rodolfo Thome[b], Guang-Xian Zhang[b*], Cun-gen Ma[a,c*]

[a] Institute of Brain Science, Datong key Laboratory of Molecular and Cell Immunology, Shanxi Datong University, Datong, 037009, China

[b] Department of Neurology, Thomas Jefferson University, Philadelphia, PA 19107, USA

[c] "2011" Collaborative Innovation Center/ Research Center of Neurobiology, Shanxi University of Traditional Chinese Medicine, Taiyuan 030024, China

[d] Institute of Neurology, Huashan Hospital, Institutes of Brain Science and State Key Laboratory of Medical Neurobiology, Fudan University, Shanghai, 200025, China

1. These authors contributed equally to this work.

Running title: ***FSD-C10 promotes neuroregeneration in EAE***

---

* Corresponding authors: Prof. Guang-Xian Zhang; Prof. Cun-gen Ma

Tel: +86-351-3179809; Fax: +86-351-2272696; Mobile: +86-18203515288.

E-mail address: guang-xian.zhang@jefferson.edu; macungen2001@163.com

Supplemental Figures

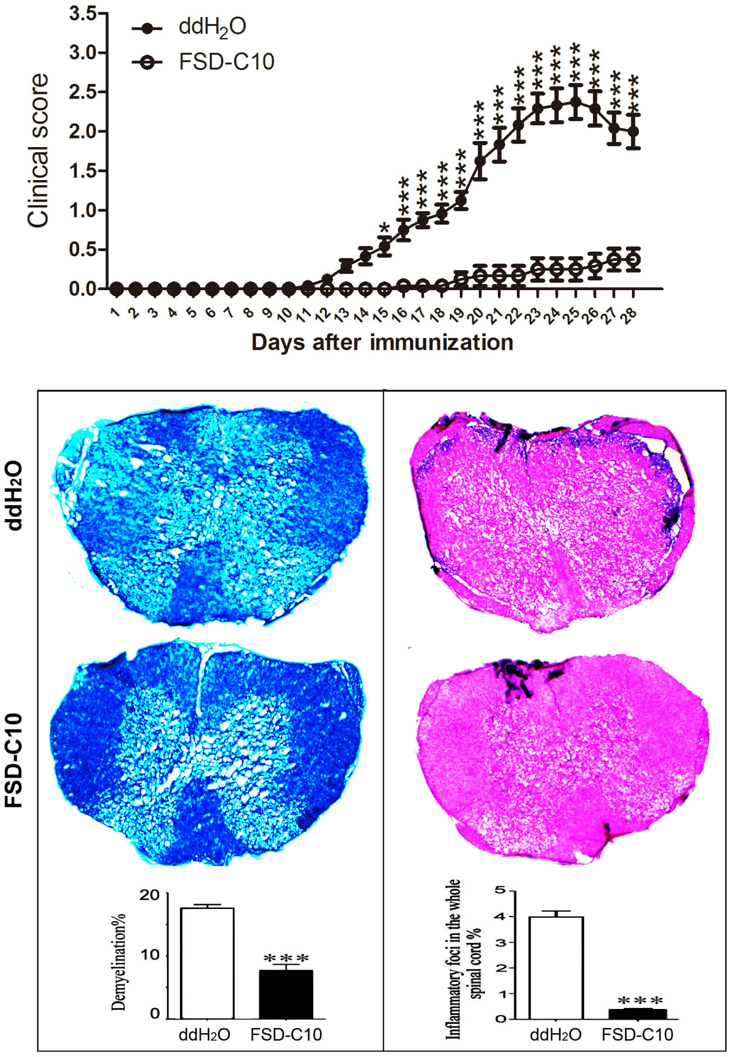

Figure S1. FSD-C10 ameliorates the severity of EAE. EAE was induced in C57BL/6 mice by active immunization with MOG_{35-55}/CFA. Mice received FSD-C10 or ddH_2O by intranasal pathway beginning on day 3 p.i. until day 27 p.i. The spinal cord of mice were harvested at day 28 post-immunization (n=12 each group). (a) The clinical score was monitored daily. The data from 12 mice per group is displayed. Mean clinical score ± SEM. *P<0.05, **P<0.01, ***P<0.001 by Mann-Whitney U test. (b) Lumbar section of spinal cord were used for inflammatory infiltration (haematoxylin & eosin staining) and demyelination (Luxol Fast Blue staining) (n=7 each group). Representative data of three independent experiments.

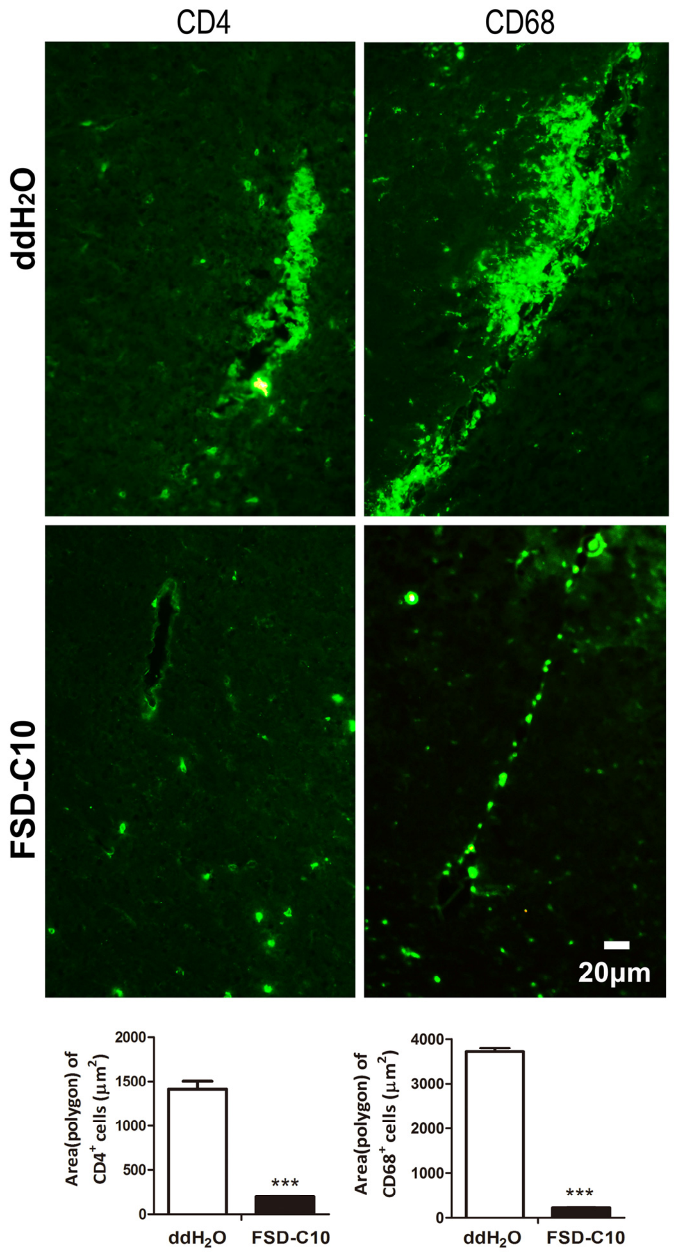

Figure S2. Reduced immune cells in FSD-C10 treated mice

Brain and spinal cords were harvested from mice described in Figure S1 at day 28 p.i. Brains were harvested for immunostaining with antibodies against CD4 and CD68. Representative microphotographs for the number of CD4 T cells and CD68 macrophages in brains and quantitative analysis of brains. Quantitative analyses represent mean ± SEM (n=7 each group). ***P<0.001. Representative data of three independent experiments.

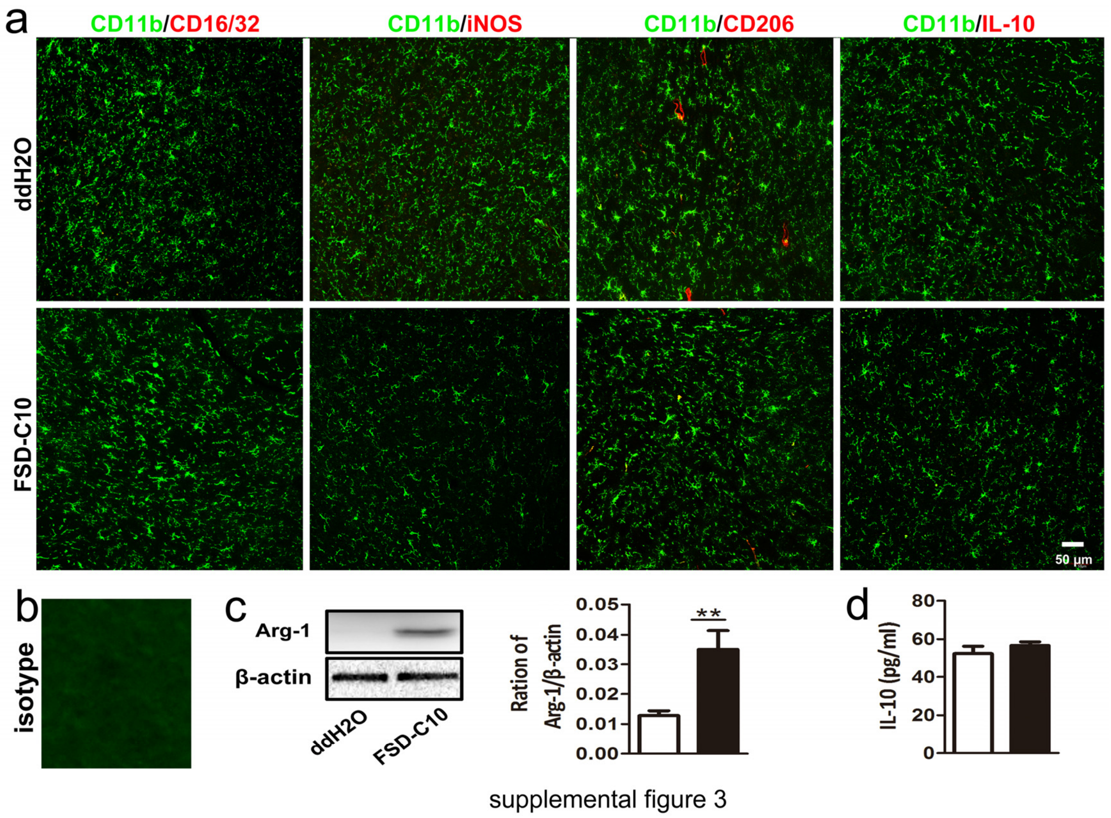

Figure S3. Intranasal FSD-C10 treatment did not affect the microglia phenotype in normal mice

Normal mice (n=6 each group) received FSD-C10 or ddH$_2$O by intranasal pathway for consecutive 25 days. Brains were harvested from these mice (a) Brains were immunostained with CD11b antibody for analyzing the functional states. Expression of M1 markers (CD16/32 and iNOS) and M2 markers (CD206 and IL-10) in CD11b$^+$ cells was determined by immunohistochemistry. (b) isotype immunofluorescence. (c) Expression of Arg-1 was measured by Western blot. (d) IL-10 expression was measured by ELISA (n=3 each group for brain section staining, and n=3 each group for Western blot and ELISA). **P< 0.01. One representative of three experiments is shown.
